# Human blood monocytes support persistence, but not replication of the intracellular pathogen *C. pneumoniae*

**DOI:** 10.1186/s12865-014-0060-1

**Published:** 2014-12-09

**Authors:** Tanja Buchacher, Herbert Wiesinger-Mayr, Klemens Vierlinger, Beate M Rüger, Gerold Stanek, Michael B Fischer, Viktoria Weber

**Affiliations:** Christian Doppler Laboratory for Innovative Therapy Approaches in Sepsis, Danube University Krems, Krems, Austria; Austrian Institute of Technology, Molecular Medicine, Vienna, Austria; Department of Blood Group Serology and Transfusion Medicine, Medical University of Vienna, Vienna, Austria; Institute for Hygiene and Applied Immunology, Medical University of Vienna, Vienna, Austria; Department for Health Sciences and Biomedicine, Danube University Krems, Krems, Austria

**Keywords:** Intracellular pathogens, Endotoxin, Monocytes, Cytokines, DNA microarray

## Abstract

**Background:**

Intracellular pathogens have devised various mechanisms to subvert the host immune response in order to survive and replicate in host cells. Here, we studied the infection of human blood monocytes with the intracellular pathogen *C. pneumoniae* and the effect on cytokine and chemokine profiles in comparison to stimulation with LPS.

**Results:**

Monocytes purified from peripheral blood mononuclear cells by negative depletion were infected with *C. pneumoniae*. While immunofluorescence confirmed the presence of chlamydial lipopolysaccharide (LPS) in the cytoplasm of infected monocytes, real-time PCR did not provide evidence for replication of the intracellular pathogen. Complementary to PCR, *C. pneumoniae* infection was confirmed by an oligonucleotide DNA microarray for the detection of intracellular pathogens. Raman microspectroscopy revealed different molecular fingerprints for infected and non-infected monocytes, which were mainly due to changes in lipid and fatty acid content. Stimulation of monocytes with *C. pneumoniae* or with LPS induced similar profiles of tumor necrosis factor-alpha (TNF-α) and interleukin (IL)-6, but higher levels of IL-1β, IL-12p40 and IL-12p70 for *C. pneumoniae* which were statistically significant. *C. pneumoniae* also induced release of the chemokines MCP-1, MIP-1α and MIP-1β, and CXCL-8, which correlated with TNF-α secretion.

**Conclusion:**

Infection of human blood monocytes with intracellular pathogens triggers altered cytokine and chemokine pattern as compared to stimulation with extracellular ligands such as LPS. Complementing conventional methods, an oligonucleotide DNA microarray for the detection of intracellular pathogens as well as Raman microspectroscopy provide useful tools to trace monocyte infection.

**Electronic supplementary material:**

The online version of this article (doi:10.1186/s12865-014-0060-1) contains supplementary material, which is available to authorized users.

## Background

Microbial pathogens have developed various strategies to escape and alter host immunity to favor their survival within the host [1,2]. Intracellular pathogens, in particular, use host cells as a replicating niche, and their release from infected cells with subsequent infection of new cells may contribute to dissemination and persistence of infection.

*Chlamydia pneumoniae* is a Gram-negative, obligate intracellular bacterium causing respiratory infections, such as acute pneumonia, bronchitis, and sinusitis [3]. Furthermore, there is a growing body of evidence for association of persistent *C. pneumoniae* infections with a range of chronic diseases, such as atherosclerosis, asthma, arthritis, multiple sclerosis, Alzheimer’s disease, and osteoporosis [4-10]. *Chlamydiae* exhibit a dimorphic life cycle with extracellular, infectious elementary bodies (EBs), and intracellular, non-infectious reticulate bodies (RBs) [11,12]. Transition into a state of persistence can be induced *in vitro* by factors such as penicillin, starvation, or maturation of the host cell. Among the susceptible host cells are the mucosal and vascular endothelium, smooth muscle cells, circulating monocytes, and tissue-specific macrophages [13]. *C. pneumoniae* can induce monocyte inflammatory cascades and modulate cellular lipid metabolism [14]. Human monocyte cell lines have been shown to transfer the pathogen to endothelial cells *in vitro* [15-17], and several lines of evidence propose a role of circulating monocytes as vehicle of its vascular dissemination [18]. Monocytes may traffic *C. pneumoniae* across the blood-brain-barrier, shed them in the central nervous system, and induce neuroinflammation [19,20].

The detection of pathogens within immune cells is challenging, and their intracellular growth may prevent correct diagnosis and appropriate treatment. The value of blood cultures as a general diagnostic tool for pathogen detection is limited due to delayed availability of results and poor sensitivity for fastidious pathogens [21].

Here, we established an *in vitro* model for the infection of immune cells with intracellular pathogens and investigated cytokine and chemokine release from human blood derived monocytes infected with *C. pneumoniae*. To trace monocyte infection, we applied a combination of methods, namely immunofluorescence and real-time PCR (RT-PCR) as well as an oligonucleotide DNA microarray for intracellular pathogens. Finally, we used Raman microspectroscopy to identify infected monocytes based on altered biomolecule fingerprints.

## Results

### Detection of *C. pneumoniae* by immunofluorescence

**Figure 1:**
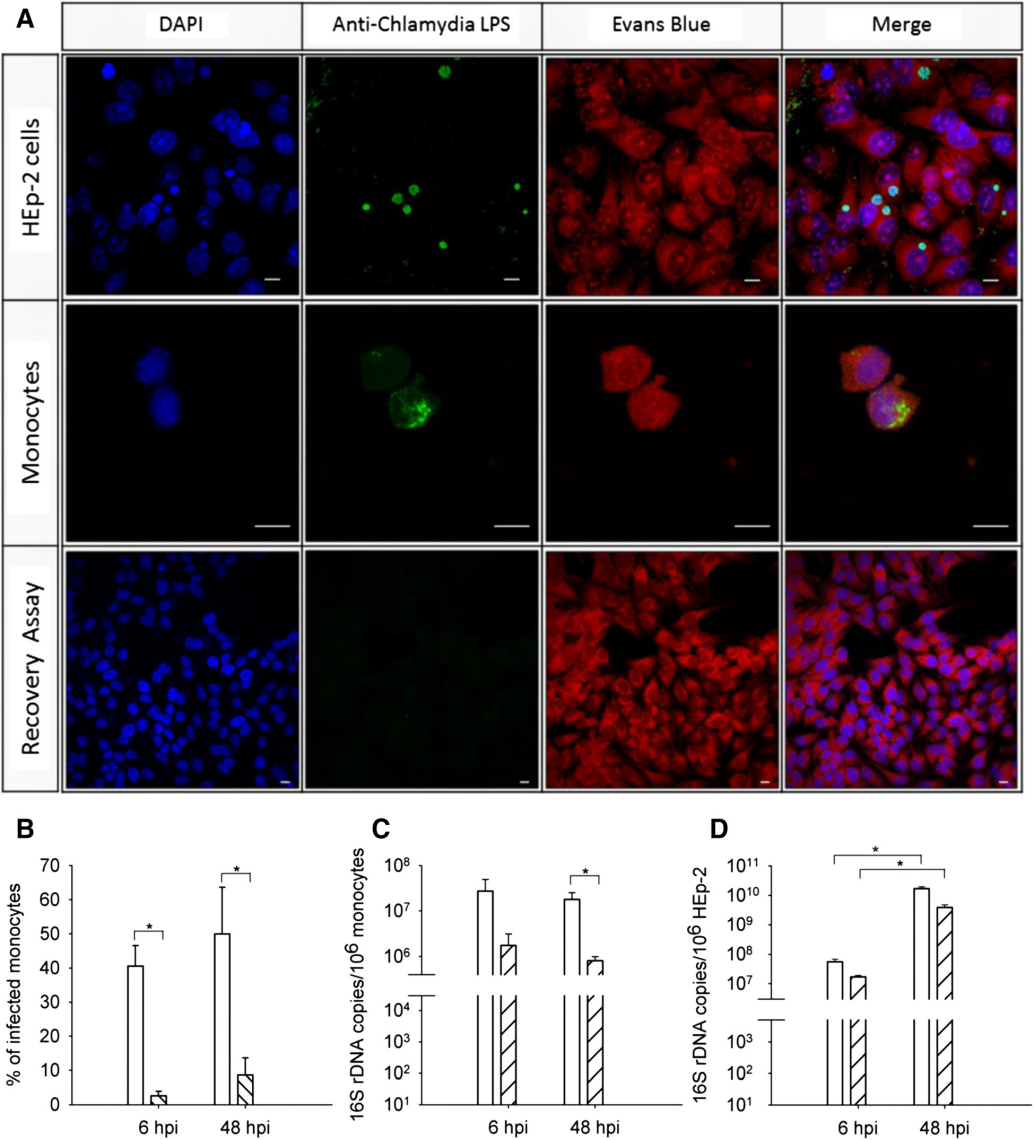
**Detection of** ***C. pneumoniae*** **in HEp-2 cells and monocytes using immunofluorescence microscopy and RT-PCR. A)** *C. pneumoniae* (green) was detected in HEp-2 cells (upper panel) and in adherent monocytes (middle panel) at 48 h post infection. Recovery of *C. pneumoniae* was evaluated by recultivating disrupted monocytes at 48 h post infection in HEp-2 cells (lower panel). Cells were counterstained with Evans Blue (red), and DNA was visualized with DAPI (blue). Chlamydial inclusions are clearly demarcated in HEp-2 cells, but appear diffuse in monocytes (scale bar = 10 μm). **B)** Monocyte infection was performed using 2 × 10^4^ IFU (open bars) and 2 × 10^3^ IFU (hatched bars) per 2 × 10^5^ monocytes, respectively, and monocyte infection rates were determined by immunofluorescence. **C)** *C. pneumoniae* 16S rDNA copies in monocytes and **D)** HEp-2 cells infected with 2 × 10^4^ IFU (open bars), 2 × 10^3^ IFU (hatched bars) and uninfected cells (grey bars) were quantified at 6 and 48 h post infection by RT-PCR. Concentrations are expressed as mean ± SD for 3 independent experiments. *P ≤ 0.05

*C. pneumoniae* was grown in HEp-2 cells, which are established for *Chlamydia* propagation [22]. Cells were lysed at 72 h post infection, and fractions of chlamydial elementary bodies were isolated and used to infect adherent monocytes. Infection was performed using 2 × 10^3^ and 2 × 10^4^ inclusion forming units (IFU) per 2 × 10^5^ monocytes, further designated as high and low dose protocols, respectively. Mature chlamydial inclusions co-localized with LPS in infected HEp-2 cells, and chlamydial DNA could be visualized at 48 h post infection (Figure 1A, upper panel). In contrast to the clearly bordered chlamydial inclusions observed in HEp-2 cells, chlamydial LPS appeared in clusters of vesicles in the cytoplasm of monocytes (Figure 1A, middle panel). Reinfection of fresh HEp-2 cells with lysates derived from infected monocytes revealed no chlamydial inclusions (Figure 1A, lower panel), indicating the absence of viable pathogen. Monocyte infection rates as determined by immunofluorescence were 3–9% for the low dose vs. 41–50% for the high dose infection (Figure 1B).

### Detection of *C. pneumoniae* by DNA-microarray and RT- PCR

Two DNA-based methods, RT-PCR and a novel DNA microarray, were employed to monitor *C. pneumoniae* infection of monocytes. RT-PCR revealed no significant changes in copy numbers of the intracellular pathogen over time (Figure 1C). No chlamydial DNA was detected in uninfected monocytes. In contrast, HEp-2 cells infected with *C. pneumoniae* exhibited an increase in the copy number of the 16S rDNA gene transcripts after 48 h of infection (Figure 1D).

**Figure 2:**
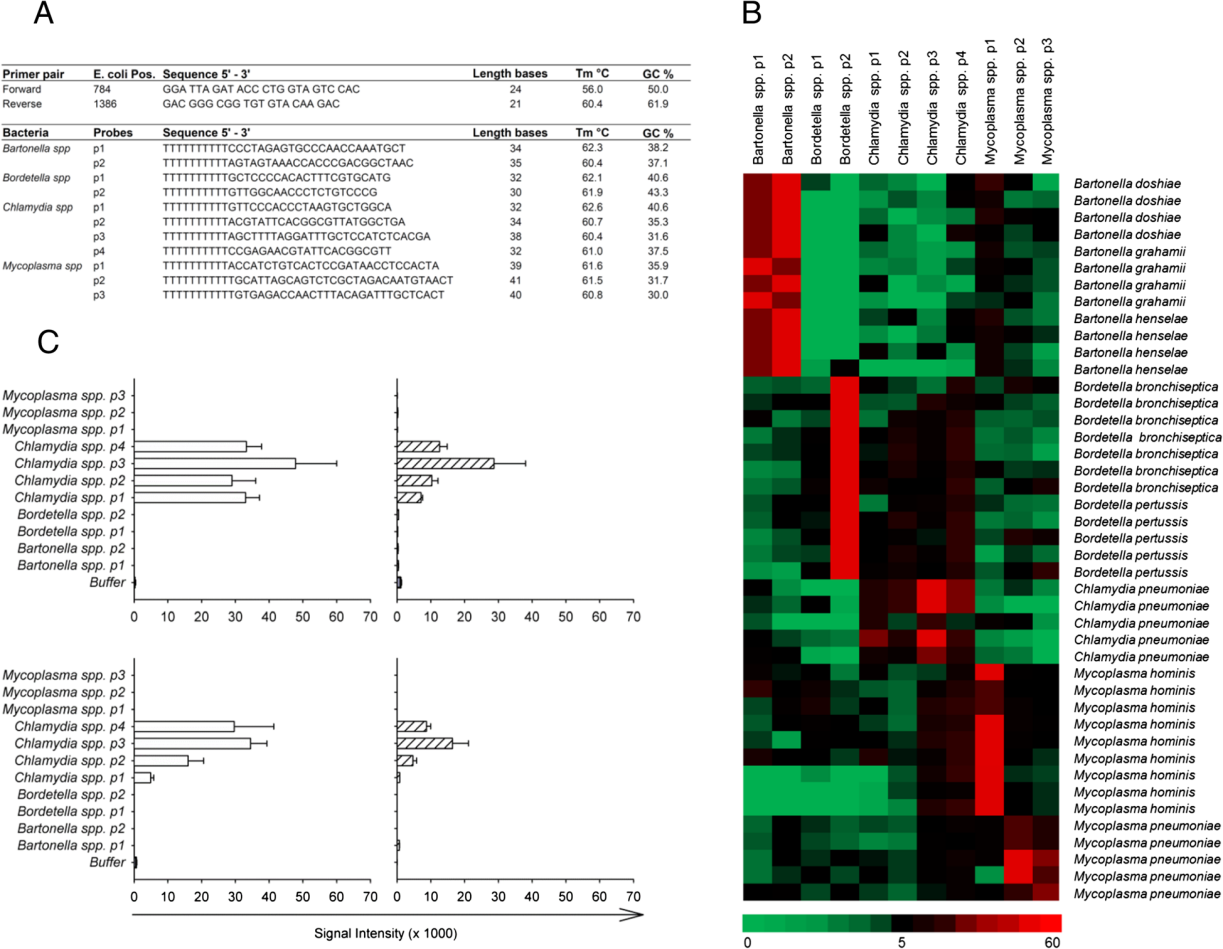
**Detection of** ***C. pneumoniae*** **by a DNA microarray for intracellular pathogens. A)** Hybridization results were obtained with genus-specific probes (p) for selected intracellular pathogens. **B)** Results for all hybridization experiments are represented as a heatmap. Columns correspond to probes and rows to hybridizations. Colors correspond to signal values (green: no signal; black: weak signal; red: strong signal). **C)** Specific hybridization patterns were obtained for monocytes infected with 2 × 10^4^ IFU (open bars) and 2 × 10^3^ IFU (hatched bars) per 2 × 10^5^ monocytes after 6 h (upper panel) and 48 h (lower panel) post infection with *Chlamydia* spp. probes. Error bars represent the mean of 4 replicate spots on the microarray. The figures show one representative microarray analysis (n = 3).

An oligonucleotide microarray was developed for the simultaneous detection of *Bartonella, Bordetella, Chlamydia* and *Mycoplasma* as described in Materials and Methods. The sequence of the chosen primer pair and oligonucleotide probes are summarized in Figure 2A. Red spots on the heatmap (Figure 2B) indicate strong hybridization of the selected oligonucleotide probes with their complementary PCR products, demonstrating the specificity of the microarray. Certain probes, such as *Chlamydia* spp. p4 showed weak cross-hybridization to *Mycoplasma* and *Bordetella*. To classify a hybridization pattern, the nearest shrunken centroid classification was used and validated in a leave-out cross-validation approach [23]. Relying on this analysis, the assay specificity for all 43 hybridizations was determined to be 100% at genus level and more than 84.1% at species level. Using this oligonucleotide microarray, DNA extracted from monocytes yielded a positive signal at 6 and 48 h post infection for the high and low dose protocol, respectively (Figure 2C).

### Detection of modified biomolecule patterns by Raman spectroscopy

**Figure 3:**
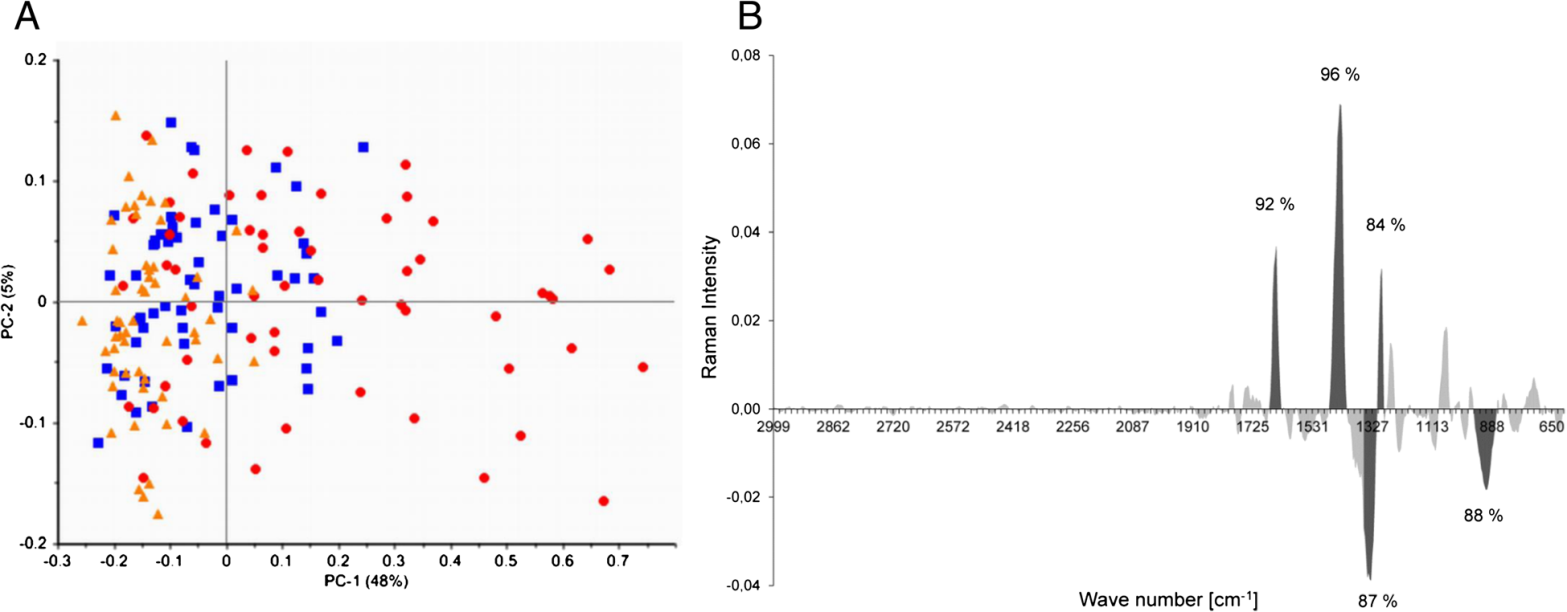
**Raman Microspectroscopy of monocytes infected with** ***C. pneumoniae*** **. A)** Score plot based on Principal Component Analysis (PCA). Monocytes infected with *C. pneumoniae* for 48 h (red dots) were separated from uninfected cells (blue dots), whereas no difference was observed after 6 h of infection (orange dots) compared to control. **B)** Mean spectra plot of PC-1 indicating the wavenumber regions which were mainly responsible for the differences between uninfected and infected monocytes after 48 h. Peaks shown in dark grey correspond to the wave number regions which are mainly responsible for data separation. The percentage of data defining the separation is indicated.

We recorded Raman spectra for monocytes infected with *C. pneumoniae* and for uninfected monocytes. The scores plot resulting after Principal Component Analysis (PCA) showed two clusters for uninfected and infected monocytes after 48 h, which can be separated on PC1 (48%), while no separation was seen after 6 h of infection (Figure 3A). Data of infected and uninfected cells after 48 h were compared in a mean spectra plot (Figure 3B) and certain wave number areas specific for infection were re-analyzed by PCA for clear separation (percentage value on PC1 > 48%). According to this analysis, differences between infected monocytes and uninfected control were found in wavenumber regions 1645–1660 cm^−1^, 1430–1451 cm^−1^, 1327–1356 cm^−1^, 1290–1306 cm^−1^, and 888–934 cm^−1^, indicating changes in lipids, fatty acids, and nucleic acids in infected monocytes [24].

### Cytokine release from infected monocytes

**Figure 4:**
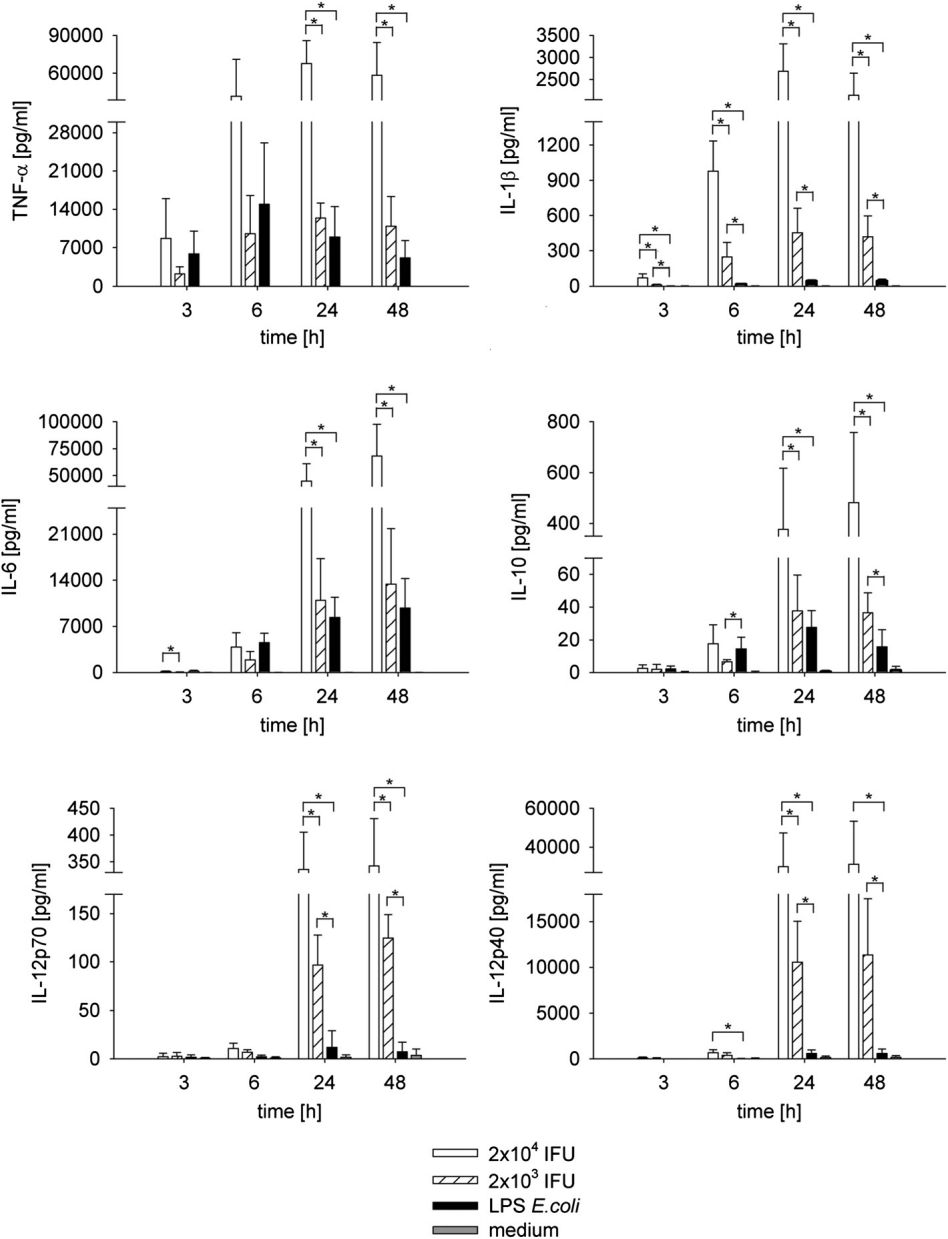
**Cytokine induction in monocytes infected with** ***C. pneumoniae*** **or stimulated with LPS from** ***E. coli.*** Human adherent monocytes (2 × 10^5^/well of 24 well plates) were infected with 2 × 10^4^ (open bars) or 2 × 10^3^ (hatched bars) inclusion forming units of *C. pneumoniae* or stimulated with 1 ng/ml LPS (black bars) for 3, 6, 24, and 48 h. Medium alone served as negative control (grey bars). Mock controls did not differ from the values obtained for “medium alone”. Concentrations are expressed as mean ± SD for 3 independent experiments. *P ≤ 0.05.

Cytokine release was assessed after monocyte infection with *C. pneumoniae* (Figure 4). Levels of tumor necrosis factor-alpha (TNF-α) increased over 6 h after post infection with low dose *C. pneumoniae* or LPS and remained stable or slightly decreased at later time points. High dose *C. pneumoniae* induced an analogous kinetic pattern of TNF-α release with approximately five-fold higher TNF-α concentrations. Secretion of IL-6 started at 6 h post infection for all stimuli with a continuous increase over 48 h. High dose *C. pneumoniae* yielded about five-fold higher IL-6 concentrations as compared to the lower dose.

While similar levels of TNF-α and IL-6 were released after infection with low dose *C. pneumoniae* or stimulation with 1 ng/mL LPS, low dose *C. pneumoniae* induced higher levels of IL-1β, IL-12p40 and IL-12p70 over time than LPS, which was shown to be statistically significant (Figure 4). Noteworthy, levels of IL-12p40 in the supernatant were about ten-fold higher than levels of IL-12p70, demonstrating that production of each of the subunits of IL-12 is independently regulated. The anti-inflammatory mediator IL-10 started to increase at 6 h post infection for all stimuli, but showed different kinetics for low dose *C. pneumoniae* and LPS. LPS yielded significantly higher levels of IL-10 than low dose *C. pneumoniae* at 6 h after stimulation, but this ratio was reversed over time, resulting in significantly higher IL-10 levels for low dose *C. pneumoniae* as compared to LPS after 48 h. Cytokine levels released from mock-infected monocytes remained below the detection limit and did not differ from uninfected controls (medium alone).

### Chemokine release from infected monocytes

**Figure 5:**
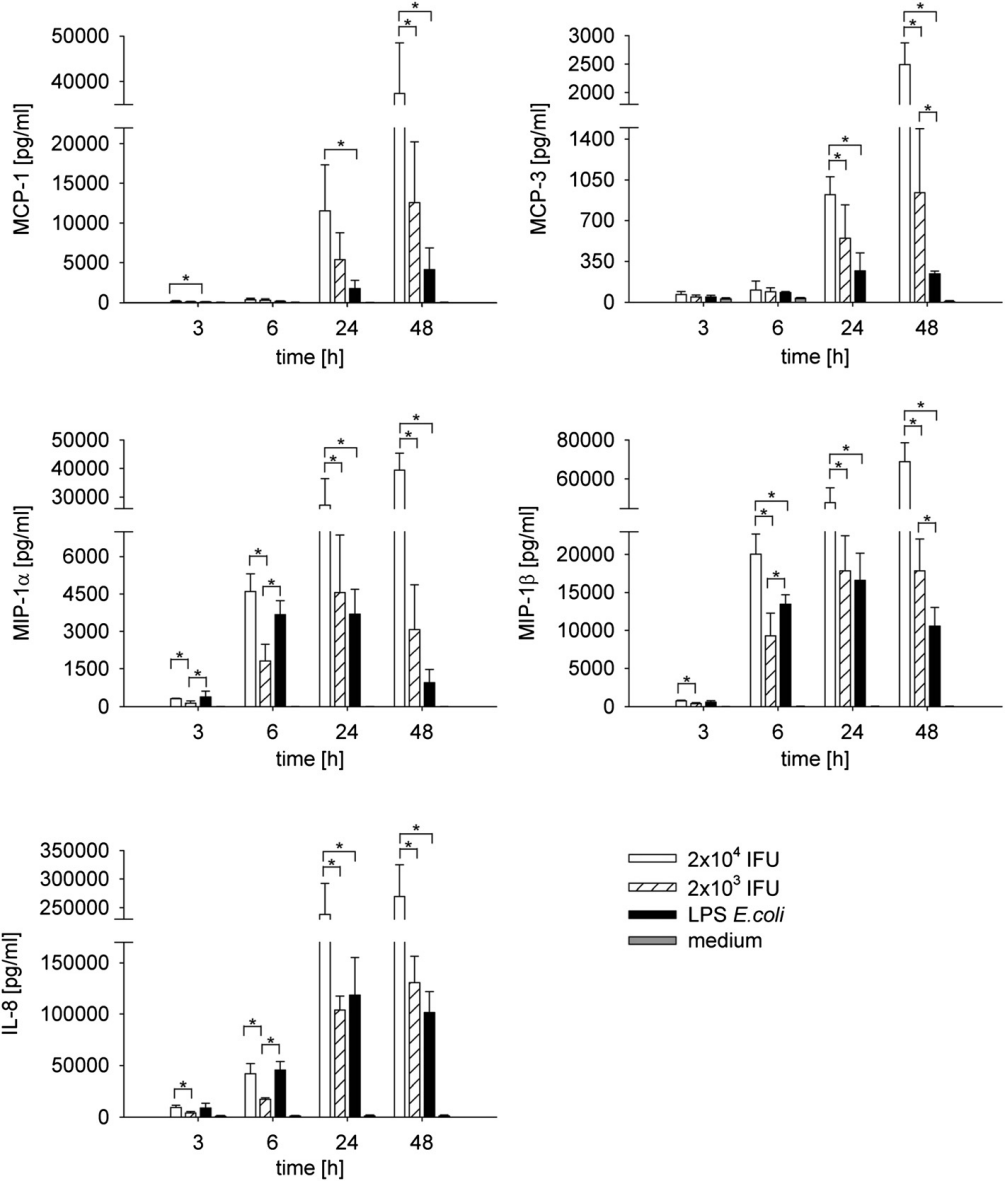
**Chemokine induction in monocytes infected with** ***C. pneumoniae*** **or stimulated with LPS from** ***E. coli*** **.** Human adherent monocytes (2 × 10^5^/well of 24 well plates) were infected with 2 × 10^4^ (open bars) or 2 × 10^3^ (hatched bars) inclusion forming units of *C. pneumoniae* or stimulated with 1 ng/ml LPS (black bars) for 3, 6, 24 and 48 h. Medium alone served as negative control (grey bars). Mock controls did not differ from the values obtained for “medium alone”. Concentrations are expressed as mean ± SD for 3 independent experiments. *P ≤ 0.05.

Secretion of monocyte chemotactic protein (MCP)-1 and MCP-3 started at 24 h after monocyte infection with *C. pneumoniae* and continued to increase for up to 48 h. LPS induced significantly lower amounts of both chemokines over time (Figure 5). Macrophage inflammatory protein (MIP)-1α and MIP-1β release started at 6 h after monocyte infection with *C. pneumoniae* and continued to increase for up to 48 h, while MIP-1 peaked at 24 h after stimulation with LPS and started to decrease thereafter. An analogous pattern was seen for IL-8 (CXCL-8), which increased continuously over 48 h after monocyte infection with *C. pneumoniae*, while levels peaked at 24 h upon LPS stimulation. Again, chemokine levels released from mock-infected monocytes remained below the detection limit.

## Discussion

Monocytes and monocyte-derived macrophages are supposed to act as vectors for the systemic dissemination of *C. pneumoniae* [25]. Here, we established an *in vitro* model to study the activation of human blood monocytes with *C. pneumoniae* and to examine immune mediator profiles secreted by infected monocytes. To detect intracellular pathogen and to analyze the potential ability of monocytes to support *Chlamydia* replication, we applied a combination of RT-PCR and immunofluorescence as well as an oligonucleotide DNA microarray for intracellular pathogens. Immunofluorescence revealed numerous small vesicles containing clusters of chlamydial LPS in the cytoplasm of infected monocytes, but it failed to detect chlamydial DNA or typical chlamydial inclusions. HEp-2 cells, which were used to propagate *C. pneumoniae*, in contrast, allowed for the formation and growth of inclusions containing chlamydial DNA and LPS. RT-PCR did not reveal significant changes in copy numbers of the intracellular pathogen over time, which indicates persistence but not replication of internalized *C. pneumoniae* in monocytes under these experimental conditions. In line with this finding, no viable *C. pneumoniae* could be recovered in HEp-2 cells upon re-infection with lysates from monocytes infected with *C. pneumoniae* for 48 h. This observation is in accordance with previously published data showing that *C. pneumoniae* does not replicate in freshly isolated monocytes, while monocyte-derived macrophages cultured for several days support the growth of chlamydial progeny [26,27]. Consistently, a comparative evaluation of monocyte infection with *C. pneumoniae* [28] showed a drastically reduced infectivity of *C. pneumoniae* in human monocytes at 24 and 48 h after infection, and no infective *C. pneumoniae* was detectable at later time points.

Complementary to immunofluorescence and RT-PCR, we confirmed the presence of *C. pneumoniae* in infected monocytes using an oligonucleotide DNA microarray for the detection of intracellular pathogens *Bartonella, Bordetella, Chlamydia* and *Mycoplasma*. DNA extracted from monocytes infected with *C. pneumoniae* yielded a positive signal at 6 and 48 h post infection for the high and low dose protocol, suggesting that the prototype DNA array has a potential to be developed into a useful diagnostic tool.

As a further method to trace monocyte infection with *C. pneumoniae*, we assessed the ability of Raman microspectroscopy to discriminate between infected monocytes and non-infected cells. Raman microspectroscopy, a combination of Raman spectroscopy and confocal microscopy, is an emerging technique to study living cells, providing fingerprints of their chemical composition. It has been applied in a recent study to characterize intracellular distribution of metabolites in *Chlamydia*-infected epithelial cells [29]. According to our results, this non-invasive technique allows for the discrimination of *C. pneumoniae* infected and non-infected monocytes, supporting its value to screen for intracellular pathogens. Of note, the changes in the wave number regions 1645–1660 cm^−1^ (unsaturated lipids) and 1327–1356 cm^−1^ (adenine) in infected monocytes in our study were comparable to data obtained in chlamydial infected epithelial cells [29].

Cytokine and chemokine release from infected monocytes were assessed in comparison to mediator release triggered by LPS. While several studies have been published on cytokine release by monocytic cell lines infected with *C. pneumoniae* [30,31], we used human blood monocytes purified from Human peripheral blood mononuclear cells (PBMCs) by negative depletion and adherence to tissue culture plates. While release of TNF-α and IL-6 did not differ significantly for stimulation with low dose *C. pneumoniae* and LPS, *C. pneumoniae* induced significantly higher levels of IL-1β, IL-12p40, and IL-12p70 than lipopolysaccharide. Secretion of IL-1β is tightly controlled and requires a first danger signal to produce pro-IL-1β and a second intracellular signal to induce caspase-1 dependent secretion of mature IL-1β [32,33]. Due to constitutive activation of caspase-1, monocytes are able to secrete IL-1β upon stimulation with single ligands, while this ability is lost after monocyte adhesion [34]. Consistently, adherent monocytes in our study failed to release significant amounts of IL-1β upon stimulation with LPS alone, whereas infection with *C. pneumoniae*, which provides both an extracellular and an intracellular stimulus, resulted in strong induction of IL-1β. The same pattern was observed for IL-12, confirming that TLR ligands alone are not sufficient to induce production of the IL-12 heterodimer [35].

Infection with *C. pneumoniae* induced substantial chemokine release from adherent monocytes in our model. MCP-1, MIP-1α, and IL-8 secretion were comparable for low-dose chlamydial infection and stimulation with LPS, correlating with similar TNF-α secretion for the two stimuli, adding evidence to the finding that MCP-1, MIP-1α and IL-8 induction depend on TNF-α [36].

## Conclusion

The intracellular pathogen *C. pneumoniae* could be detected in human blood monocytes using a combination of immunofluorescence and RT-PCR, but we found no evidence for chlamydial replication. In addition, the oligonucleotide DNA microarray for the detection of intracellular pathogens as well as Raman microspectroscopy provided robust values to track infection of immune cells with intracellular pathogens. Infection of human blood monocytes with *C. pneumoniae* resulted in cytokine and chemokine profiles which differed significantly from stimulation with LPS, underscoring the ability of intracellular pathogens to alter innate immune response and to enhance mediator release via simultaneous activation of both membrane-bound and cytosolic pattern recognition receptors.

## Materials and methods

### Cell culture media and reagents

Phosphate-buffered saline (PBS), Eagle’s minimum essential media (MEM) GlutaMAX, gentamicin/amphotericin B solution were obtained from Invitrogen (Lofer, Austria). RPMI-1640, MEM non-essential amino acid, human male AB serum (sterile-filtered), cycloheximide, 4-(2-hydroxyethyl)-1-piperazineethanesulfonic acid (HEPES), fetal bovine serum (FBS), and lipopolysaccharide (LPS) from *E. coli* (055:B5, purified by gel filtration) were purchased from Sigma-Aldrich (St Louis, MO, USA). Fluorescein isothiocyanate (FITC)-conjugated anti-CD45 monoclonal antibody (mAb), R-phycoerythrin (PE)-conjugated CD14 mAb and the respective IgG control antibodies were from Becton Dickinson (Vienna, Austria).

### Propagation of *C. pneumoniae*

*C. pneumoniae* strain CWL-029 was obtained from the American Type Culture Collection (ATCC, VR-1310) and propagated in HEp-2 cells (ATCC, CCL23) as previously reported [22,37,38]. In brief, HEp-2 cells were passaged in Eagle’s MEM GlutaMAX supplemented with 10 μg/ml gentamicin, 0.25 μg/ml amphotericin B, 1 vol% MEM non-essential amino acids and 10 vol% heat-inactivated FBS. Confluent monolayers were infected with *C. pneumoniae* and grown in the medium described above containing 1 μg/mL cycloheximide, but lacking antibiotics. Cells were spun at 1700 g for 1 h at 35°C to enhance infectivity. At 72 h post infection (hpi) at 35°C and 5% CO_2_, the cell monolayer was disrupted using a cell scraper and zirconium dioxide beads. Chlamydial EBs were obtained by sequential centrifugation of the lysates at 600 g (10 min) and at 30,000 g (1 h; 4°C). The pelleted EBs were suspended in sucrose-phosphate-glutamic acid buffer (0.2 M sucrose, 3.8 mM KH_2_PO_4_, 7.2 mM Na_2_HPO_4_, 5 mM L-glutamic acid, pH 7.4) and stored at −80°C. The number of chlamydial inclusion forming units (IFU) per mL was determined by infectivity titration of EBs in HEp-2 cells for 48 h at 35°C, followed by immunofluorescence staining as described below. To exclude mycoplasma contamination, cell culture and chlamydial stocks were regularly tested using the Venor™GeM *Mycoplasma* Detection Kit (Minerva Biolabs, Berlin, Germany).

### Isolation and culture of monocytes

PBMCs were isolated from leukocyte reduction system (LRS) chambers of a TrimaAccel® blood collector after approval by the ethics committee of the Medical University Vienna and written informed consent were obtained from all participants (ECS2177/2013). Blood from LRS chambers was diluted 1:8 (vol/vol) with PBS containing 2 mM ethylene diamine tetraacetic acid (PBS/EDTA), and PBMCs were enriched by Ficoll gradient centrifugation. Monocytes were isolated by negative depletion with the monocyte isolation kit II (Miltenyi Biotec, Bergisch Gladbach, Germany) yielding >80% CD14 positive cells as confirmed by flow cytometry. Viability was >95% as determined by exclusion of 7-ADD.

Monocytes were resuspended at a concentration of 4 × 10^5^/mL in serum-free RPMI-1640 supplemented with 20 mM HEPES and cultured as described [39]. Aliquots of 0.5 mL/well of the monocyte suspension were seeded onto 24 well flat-bottomed tissue culture plates (Corning Incorporated, NY, USA). After 3 h at 37°C, the monocyte monolayer was gently washed with serum-free RPMI-1640 to remove non-adherent cells. Adherent monocytes were kept in RPMI-1640 medium complete containing 20 mM HEPES and 10 vol% human AB serum for an additional 24 h at 37°C.

### Infection of adherent monocytes

Adherent monocytes were inoculated with 2 × 10^3^ or 2 × 10^4^ chlamydial IFU/well, respectively, or with 1 ng/mL LPS (positive control) in a final volume of 0.5 mL medium complete. For infection, cells were centrifuged 30 min at 600 g and incubated at 37°C in 5% CO_2_. Cumulative culture supernatants were collected after 3, 6, 24 and 48 h, respectively, without replacing with fresh media, centrifuged at 600 g for 5 min at 4°C, and stored at −80°C until quantification of cytokines. Mock controls were prepared following the propagation, harvest and purification procedure for EBs [40,41], but in the absence of chlamydial infection.

### Recovery assay

The monocyte monolayer exposed to *C. pneumoniae* for 6 and 48 h was washed with PBS, and cells were scraped and vortexed with zirconium dioxide beads. EBs were obtained from the lysates as described above and passaged onto HEp-2 cells. At 48 hpi, HEp-2 cells were fixed and stained for immunofluorescence as described.

### DNA isolation and quantification

Adherent monocytes infected with *C. pneumoniae* for 6 and 48 h or uninfected cells were washed with PBS and cells were counted on plates prior to DNA isolation. Total genomic DNA was isolated and purified using the QIAmp Mini DNA kit (Qiagen, Hilden, Germany). Purified DNA was quantified at 485/530 nm using the Quant-iTdsDNA HS assay and the Qubit™ fluorometer (Invitrogen, Lofer, Austria).

### Real-time quantitative PCR

*C. pneumoniae* genomes were quantified by real-time quantitative PCR, targeting a 222 bp sequence present on *Chlamydia* 16S rDNA. The oligonucleotide primers and TaqMan probes were synthesized by Microsynth AG (Balgach, Switzerland) and used as described in detail previously [42]. RT-PCR was performed in a final volume of 20 μL including 1x Master Mix and Taq polymerase (Mastermix 16S, Molzym, Bremen, Germany), forward primer (0.75 μM), reverse primer (0.75 μM), FAM-TAMRA probe (0.75 μM) and 2 ng of DNA. Amplification and detection was performed for 10 min at 95°C, followed by 50 cycles of 10 s at 95°C and 65 s at 60°C. Standards of known concentration (10^1^, 10^2^, 10^3^, 10^4^, 10^5^ and 10^6^ copies) were prepared for the 16S rDNA target gene from PCR amplified *C. pneumoniae* DNA by conventional PCR, and purified with a QIAmp Mini DNA kit, according to the manufacturer’s instructions. Samples were run in triplicate and all reactions were carried out using the iCycler IQ system (BioRad, Vienna, Austria).

### Detection of intracellular pathogens by DNA microarray

In addition to RT-PCR, a prototype oligonucleotide microarray was developed. DNA amplification and labeling was carried out with one universal primer pair targeting a specific region of the 16S rRNA gene. Hybridization was performed with novel probes for *Bartonella, Bordetella, Chlamydia and Mycoplasma*, which were designed and modified as described [23,43]. Four replicates of each probe at a concentration of 50 μM were printed onto silylated glass slides with reactive aldehyde groups (CSS-100 Silylated Slides; CEL Associates, Texas, USA) by the contact arrayer Omnigrid from GeneMachines (San Carlos, CA, USA) with MP 3 pins (TeleChem, Sunnyvale, CA, USA) leading to spot size of 100 μm. A hybridization control probe (5’ –TTA AAA CGA CGG CCA GTG AGC) was spotted on the array applying the same conditions as used for the target capture probes. DNA amplification and primer extension were performed according to [23] with modifications as described in detail in Additional file 1. Slides were scanned using an Axon Genepix 4000A microarray scanner (Axon, Union City, California) and data were analyzed as described [23].

### Raman microspectroscopy

Monocytes infected with *C. pneumoniae* (2 × 10^4^ IFU) were cultured for 6 and 48 h on glass bottom μ-slides (170 μm thickness; ibidi GmbH, Munich, Germany). Samples were fixed with 4% paraformaldehyde for 4 min and washed 3 times with PBS. Raman spectroscopy was performed by CellTool (Bernried, Germany) using the Bio-Ram® system and the Bio-Ram® software. Raman spectra of 60 cells per assay were recorded with a 785 nm laser (80 mW), applying an accumulated time of 3 × 10 sec. Data of the biologically relevant region (700–3000 cm^−1^) were pre-treated with a median filter for noise reduction, unit vector normalisation and subsequent multivariate data analysis were done with the statistical software the Unscrambler X 10.3 (Camo Software, Oslo, Norway). We performed Principle Component Analysis (PCA) using the NIPALS algorithm and cross validation, which is a common procedure for spectral data analysis.

### Quantification of cytokines and chemokines

The levels of tumor necrosis factor-alpha (TNF-α), interleukin (IL)-1β, IL-6, IL-12p70, IL-12p40, IL-10, monocyte chemotactic protein (MCP)-1 (CCL-2), MCP-3 (CCL-7), macrophage inflammatory protein (MIP)-1α (CCL-3), MIP-1β (CCL-4), and IL-8 (CXCL-8) were determined in culture supernatants using the Bio-Plex 200 system (Bio-Rad, Vienna, Austria).

### Immunofluorescence

Infected HEp-2 cells or monocytes were cultured on μ-slides (ibidi GmbH, Munich, Germany) at 6 or 48 hpi, washed with PBS and fixed in 0.5 mL of methanol for 10 min. To visualize chlamydial inclusions, cells were stained with FITC-conjugated anti-Chlamydia-LPS mAb and human cells were counterstained with Evans Blue (Pathfinder, Chlamydia Culture Confirmation System, Bio-Rad, Vienna, Austria). The cells were mounted in Fluoromount-GTM containing DAPI (Southern Biotech, Birmingham, UK) and fluorescence images were acquired with a Zeiss LSM 700 laser scanning confocal microscope (Carl Zeiss SAS, Jena, Germany) using a 40x oil objective/1.3 NA or 63x oil objective/1.4 NA.

### Flow cytometry

Purity and viability of monocytes were examined by determination of CD14 positive cells and 7-aminoactinomycin D (7-AAD) exclusion. Monocytes were stained with unconjugated 7-AAD (BioLegend, Fell, Germany), FITC-conjugated anti-CD45 and PE-conjugated CD14 or with the respective IgG control antibody in PBS supplemented with 2 vol% FBS, 0.1 w% sodium azide at 4°C for 30 min. After one washing step, cells were analyzed on a Beckman Coulter FC 500 flow cytometer (Beckman Coulter, Vienna, Austria) and data were analyzed using the FlowJo software (Tree Star Inc, Ashland, OR).

### Statistical analysis

Statistical analysis was performed using the software package SPSS Statistics for Windows, version 18.0 (SPSS Inc., Chicago, Illinois, USA). When comparing two groups, data were analyzed by the nonparametric Wilcoxon rank sum test. Data are expressed as means ± SD. Significance was accepted at P ≤ 0.05.

## Additional file

Additional file 1:
**Materials and methods of the DNA microarray analysis.**
