# Supplementary material for: Human blood monocytes support persistence, but not replication of the intracellular pathogen C. pneumoniae
Source: BMC Immunol. 2014 Dec 9;15:60. doi: 10.1186/s12865-014-0060-1 (PMC4268907; doi:10.1186/s12865-014-0060-1)
Supplement: Additional file 1: — Materials and methods of the DNA microarray analysis. [file 12865_2014_60_MOESM1_ESM.pdf]

Additional file 1: Materials and methods of the DNA microarray analysis.

DNA amplification was carried out in 25 µl PCR reaction, including 1x master mix and MolTaq polymerase (Molzym16S Basic, Molzym, Bremen, Germany), one universal primer pair (forward 784 and reverse 1386 primer, each 0,12 µM) and 1 ng per µl of DNA extract. PCR cycling included an initial denaturation step at 95°C, 5 min, followed by 40 cycles of 95°C for 1 min, 60°C for 1 min, 72°C for 1 min, and a termination step at 72°C for 10 min using a GeneAmp® PCR System 2700 thermal cycler (Life Technologies, Vienna, Austria). Successful amplification was confirmed by analyzing the PCR products on a 1.5 % agarose gel with Sybr Safe in 1x TBE buffer (Sigma Aldrich, St Louis, MO, USA). For labeling, the primer extension method was used and a final primer extension reaction mixture of 40 µl containing 7µl of PCR product, 0.9 µM forward primer 784, 0.08 U Vent (exo^-) DNA polymerase (New England Bio Labs, Ipswish, UK), 1x PCR-buffer, 1 mM $MgSO_4$, 50µM dGTP, dATP, dTTP, 25µM dCTP (Roche Life Science, Vienna, Austria) and 0.06 U 5´-Propagylamino-dCTP-Atto532 (MoBiTec, Goettingen, Germany). PCR cycling conditions consisted of 95°C for 3 min, followed 25 cycles at 95°C, 58°C and 72°C each 20 sec and a final extension step for 3 min at 72°C. Prior to hybridization, the spotted slides were blocked with 3 M urea and 0.1% (w/v) SDS for 30 min at room temperature, washed, dried by centrifugation and covered with HybriWell™ (Grace Bio Labs, Blend, OR, USA). Hybridization mixture, containing 40 µl prewarmed Express Hyb hybridization solution (Clontech, Mountain View, CA, USA) and 40 µl labeled DNA product and a hybridization control (0.125 µM Bsrev-Cy5-AAGCTCACTGGCCGTCGTTTTAAA) was denaturated at 95°C for 3 min, prior transferring 50 µl of the suspension to the pre-treated microarray surface. Hybridization was carried out at 65°C for 1 h in a humidity chamber. Afterwards, the hybriwells were removed, and the slides were washed and dried.
